# Quantum Mechanical-Based Stability Evaluation of Crystal Structures for HIV-Targeted Drug Cabotegravir

**DOI:** 10.3390/molecules26237178

**Published:** 2021-11-26

**Authors:** Yanqiang Han, Hongyuan Luo, Qianqian Lu, Zeying Liu, Jinyun Liu, Jiarui Zhang, Zhiyun Wei, Jinjin Li

**Affiliations:** 1 Shanghai Key Laboratory of Maternal Fetal Medicine, Shanghai First Maternity and Infant Hospital, School of Medicine, Tongji University, Shanghai 200092, China; hanyanqiang@sjtu.edu.cn (Y.H.); 2031246@tongji.edu.cn (Z.L.); 2 Key Laboratory for Thin Film and Microfabrication of Ministry of Education, Department of Micro/Nano-electronics, Shanghai Jiao Tong University, Shanghai 200240, China; joker_luo@sjtu.edu.cn (H.L.); luqianqian_studying@sjtu.edu.cn (Q.L.); 3 Key Laboratory of Functional Molecular Solids of the Ministry of Education, Anhui Provincial Engineering Laboratory for New-Energy Vehicle Battery Energy-Storage Materials, School of Chemistry and Materials Science, Anhui Normal University, Wuhu 241002, China; 4 Division of Computational Biomedicine, Boston University School of Medicine, Boston, MA 02118, USA; zjr@bu.edu

**Keywords:** ab initio calculation, HIV pre-exposure prophylaxis drug, cabotegravir, crystal stability determination

## Abstract

The long-acting parenteral formulation of the HIV integrase inhibitor cabotegravir (GSK744) is currently being developed to prevent HIV infections, benefiting from infrequent dosing and high efficacy. The crystal structure can affect the bioavailability and efficacy of cabotegravir. However, the stability determination of crystal structures of GSK744 have remained a challenge. Here, we introduced an ab initio protocol to determine the stability of the crystal structures of pharmaceutical molecules, which were obtained from crystal structure prediction process starting from the molecular diagram. Using GSK744 as a case study, the ab initio predicted that Gibbs free energy provides reliable further refinement of the predicted crystal structures and presents its capability for becoming a crystal stability determination approach in the future. The proposed work can assist in the comprehensive screening of pharmaceutical design and can provide structural predictions and stability evaluation for pharmaceutical crystals.

## 1. Introduction

With the rapid development of modern medicine, the HIV-infected population is gradually decreasing, and an alternative effort—namely, pre-exposure prophylaxis (PrEP)—is now emerging as a new approach in the global battle against HIV [1]. However, the currently approved HIV PrEP formulations require daily dosing to maintain an effective drug concentration in the blood and tissues [2]. To overcome this issue, an HIV integrase inhibitor, cabotegravir (also known as GSK744) [3,4], was developed recently for treating HIV infection and provides infrequent dosing and long-acting protection [5,6]. Based on the experimental methodologies, modest to extremely high levels of stereochemical control can be investigated depending on the ring size and position of the stereocenter, but the microstructure perspective exploration of GSK744 with quantitative accuracy is still missing. Specifically, only one crystal structure of this class of agents, dolutegravir in its sodium salt form, is included in the database of the Cambridge Crystallographic Data Center (CCDC).

Pioneers used powder X-ray (or neutron) diffraction (XRD) [7,8] and solid-state nuclear magnetic resonance (NMR) [9,10,11,12] to determine the unknown structure of compounds based on their chemical compositions. While potentially powerful, these experimental methods are usually very demanding. Recent progress in the development of crystal structure prediction (CSP) helps to characterize the observed solid forms [13,14,15,16]. Especially on the flexible pharmaceutical-like organic molecules, such as olanzapine [17,18,19] and axitinib [20], a set of plausible crystal structures have been studied using successful methodologies for exploring the lattice energy landscape. Additionally, the research by Sally Price’s group utilizes a semi-empirical tight-binding method to perform relatively reliable pre-relaxation of crystal structures for flexible molecules [13]. Manolis and co-workers investigated the ability of ab initio CSP techniques to identify the polymorph of 5-methyl-2-[(2-nitrophenyl)amino]-3-thiophenecarbonitrile, named as ROY because of its red, orange, and yellow colors [21]. Because most organic molecules are not rigid with some torsion angles, they are likely to contribute intermolecular interactions to the lattice energy. Thus, the CSP of flexible molecules needs an accurate balancing of the intermolecular and intramolecular forces to get the optimal arrangements [22]. It is notable that molecule-specific force-fields for pharmaceuticals can significantly reduce the number of low-energy crystal structure calculations needed, as the approach utilized by Avant-Garde Materials Simulation [23]. Neumann et al. combined CSP and high-pressure crystallization in rational pharmaceutical polymorphs, thereby providing an excellent recipe for crystallization experiments [24]. The CSP methodology is advancing rapidly. The “PROM” search approach [25] adds crystallographic symmetry elements into structures and screens the structures based on a simple force field. Moreover, MOLPAK [26] seeks packed structures in common types of coordination by a pseudo-hard sphere model. More extensive CSP methods, such as GRACE [27], are based on monitoring the appearance rate of new structures to converge the search using molecule-specific force-field models. Both of them apply to multi-component systems and flexible molecules. Following the structure generation of the CSP process, the stability or probability of each structure is another main task, which is often evaluated by lattice energy. Additionally, Gibbs free energy has been considered an essential physical quantity for stability evaluation.

The structures screened by MOLPAK are subjected to a preliminary, low-precision calculation of the lattice energy. Gibbs free energy is a significant parameter not only for crystal structure but also for crystal morphology. Spiral growth is a kinetic process, and the measurement of crystal growth is often based on thermodynamic properties. During crystal growth, when new molecules are added, the critical length becomes longer and the Gibbs free energy of the crystal changes [28]. If the Gibbs free energy of the crystal is not changed by the addition of extra molecules, the crystal will stop growing on this edge.

**Figure 1:**
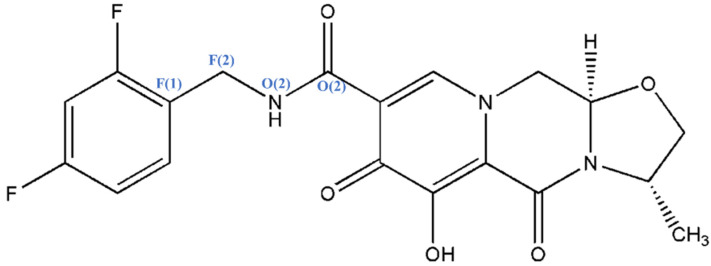
Chemical structure of GSK744, which has a formula of C_19_H_17_F_2_N_3_O_5_.

Here, we utilized MOLPAK software as the initial search tool for providing a 3D map of a minimum unit cell based on the orientation function of molecules and generated a few thousand plausible candidates. Based on the calculation of lattice energy, CSP was commonly paired with XRD [29] to identify different polymorphs of pharmaceutical and biological molecules but had a restriction to correlate the predicted structures having lowest-energies within about 10 kJ·mol^−1^; therefore, it could not reach the desired accuracy and provide reliable candidates [17,30,31]. Making use of the CSP method combining re-optimization of the most promising structures and energetic re-ranking results significantly resulted in the most possible packing of the predicted molecule [32,33,34]. In this work, we used Gibbs free energy rather than lattice energy to evaluate the structural stability of GSK744, which takes into account multiple factors, such as entropy, temperature, and polarization effects and which is more accurate when evaluating the stability of a crystal structure. Figure 1 shows the chemical structure of GSK744, which is a large drug molecule containing 46 atoms (with a molecular weight of 405 g/mol). Conventional high-level quantum chemistry methods with a highly accurate benchmark were used to perform the required calculations of target molecules that required great computational cost. The CSP combining density functional theory (DFT) is already used for large molecules (up to at least 600 Da) [35,36,37]. Thus, in this work, Gibbs free energy calculations were performed using density functional theory (DFT) along with the embedded fragment quantum mechanical (EF-QM) method. The fragment QM method can divide the internal energy per unit cell of a crystal into a proper combination of the energies of monomers and dimers, which are embedded in the electrostatic field of the rest of the crystalline environment and which treat the macromolecules effectively [38,39,40,41,42,43,44]. The interaction energies between two fragments within a distance threshold were calculated by QM, while the interaction between two long-range interacting fragments was treated by charge–charge Coulomb interactions [45]. 

**Figure 2:**
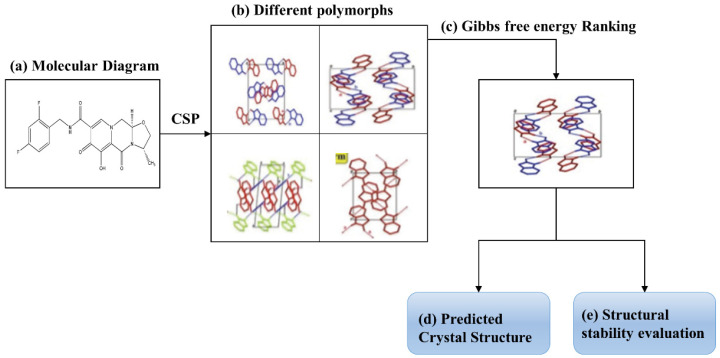
Flow chart of the introduced ab initio protocol for predicting the most likely stable crystal structure and morphology of molecular crystal. (**a**) Molecular diagram of a molecule. (**b**) Obtained polymorphs of a molecular crystal based on the CSP method. (**c**) Gibbs free energy ranking of the polymorphs. (**d**) Predicted most likely stable crystal structure. (**e**) Stability evaluation of crystal structures.

Figure 2 shows the flow chart of the introduced ab initio protocol for predicting the most likely stable crystal structure of a molecule and for determining the stability of crystal structures. The protocol started with the molecular diagram and performed CSP to generate different polymorphs of the molecule. Then, the Gibbs free energies of these polymorphic structures were calculated by the EF-QM method, and the optimal structure was determined at the bottom of the Gibbs free energy landscape. Concomitantly, the stability of crystal structures can be evaluated based on the Gibbs free energy calculations.

## 2. Results and Discussion

### 2.1. Conformational Search

**Figure 3:**
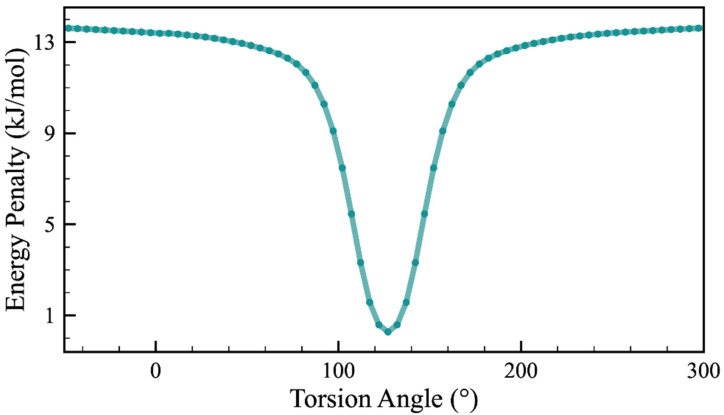
Relative energy penalty against the selected torsion angle (F(1)-F(2)-O(1)-O(2)) from −50° to 310°, generated from the PES scan.

Small conformational variations of a flexible molecule, e.g., adjustments in torsion angles by a few degrees, can change the final energy landscape significantly, which brings more challenges into a CSP study [46,47,48]. As a flexible molecule with a chiral center, minor changes in GSK744 conformation should be taken into account at the determination of the crystal structures. By the topology analysis of the flexible GSK744 molecule, we can easily identify one dihedral angle, the torsion angle of F(1)-F(2)-O(1)-O(2) (Figure 1), which covers the flexible middle linker region in GSK744. Thus, we preformed the potential energy surface (PES) scan for that dihedral angle to identify a self-assembling conformer with the lowest potential energy. Moreover, because the MOLPAK merely enables the handling of rigid molecules, only the one corresponding to the energy minima of PES can be the input conformer. The result of the PES scan is shown in Figure 3, suggesting that a molecule with a torsion angle of 127.1° reaches the global minimum and achieves the optimal arrangement. Thus, the obvious valley-like variations of the energy penalty indicate that conformational flexibility plays a significant role in the molecular geometry and indirectly influence the thermodynamic stability of the molecule. The conformer found in a solid could be very different from the most stable molecule. The PES scan here offers a glimpse into the molecular flexibility of the linker region and provides a reliable conformer, leading to a relatively high-quality population of CSP. 

### 2.2. Gibbs Free Energy Guided CSP

**Figure 4:**
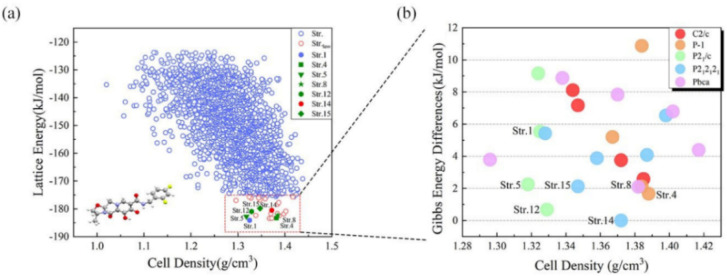
Lattice energy and Gibbs free energy landscapes for the predicted GSK744 candidates. (**a**) Lattice energies (from −120 to −190 kJ/mol) of all predicted GSK744 candidates; the blue circles are the predicted 3000 hypothetical structures, and the circles in the red rectangle are the candidates with the lower lattice energies (below −175 kJ/mol). (**b**) Gibbs free energy differences per primitive unit cell of the 24 predicted candidate structures as functions of the unit cell density. Points with the same color represent structures with the same space group. The energies of all the predicted structures are with reference to the energy of Str. 14.

In this work, a global search for the structure of GSK744 was performed with the MOLPAK package [26]. In Figure 4a, the obtained lattice energy landscape is plotted from −190 kJ/mol to −120 kJ/mol. The points in the red rectangle are the selected candidates with the lower lattice energies (below −175 kJ/mol). Above this value, there is a plethora of crystal structures whose relative lattice energies are sufficiently high to suggest that they are less likely to be viable experimental forms. In Figure 4a, the selected 24 candidates in the red rectangle (Str.1, Str.4, Str.5, etc.) come from five space groups (*P*2_1_/*c*, *P*-1, *C*2/*c*, *Pbca*, and *P*2_1_2_1_2_1_) within the energy difference of 10 kJ/mol. Table S1 in the Supplementary Materials (SM) lists the details of the lattice parameters of the 24 predicted candidates. Based on the calculation of lattice energy, Figure 4a shows that Str.1 and Str.4, having the lowest lattice energies, are possible stable crystal structures of GSK744. 

The 24 selected candidates were further evaluated using the Gibbs free energy calculation, which takes into account the entropy, temperature, and polarization effects to obtain more stable structures. We adopted DFT along with the EF-QM method to calculate the Gibbs free energies of the 24 candidates, where all crystal structures were optimized at the ωB97XD/6-31G* level. Figure 4b shows the Gibbs free energy differences between 24 candidates of GSK744, with reference to the energy of Str. 14 (−5894.675 Hartree). Str.1 and Str.4 are not at the bottom of this figure. Str.14, which is not at the bottom of the lattice energy landscape, is the structure with the lowest Gibbs free energy, indicating that Str.14 has the highest thermodynamic stability and is the most likely structural candidate of GSK744. The crystal structure of Str.14 is presented in Supplementary Materials (SM) in PDB format. Most CSP techniques generally identify the most likely crystal structure by searching for the most thermodynamically stable structure, and at least initially, most programs are based on the theory that the thermodynamic stability can be approximated by the lowest lattice energy. However, without consideration of a temperature effect, lattice energy only predicts the relative thermodynamic structures at extra-low temperatures. Gibbs free energy, on the other hand, offers an alternative and/or complementary standard for evaluating the predicted crystal structures and polymorphs discovery [17].

## 3. Methods

The ab initio CSP method was used for the structure prediction of GSK744, which started with a potential energy surface scan to obtain a favorable conformer and a global search to determine the quality of candidates from the energy landscape. The optimal crystal structure can be selected by minimizing the lattice energy. In this work, MOLPAK software was used for the global search of structures with lowest lattice energies from all space groups. For GSK744, MOLPAK generated more than 3000 crystal structures, where 24 candidates were located at the bottom of the energy landscape. The 24 candidates proceeded to the next round of Gibbs free energy calculations, which was performed by our in-house parallel execution code. In this manuscript, the proposed Gibbs free energy calculations were performed by the embedded fragment QM method, along with the DFT or the high-level second-order Møller–Plesset perturbation (MP2) theory. In this work, the DFT method was selected for QM calculation.

### 3.1. Potential Energy Surface (PES) Scan

For GSK744, as a flexible torsion molecule, the first step was to determine the preferable torsion angle with energy minima determined by PES scan. It involved a set of different rigid conformations to locate the favorable torsion angle with the energy minimum [49]. Note that each conformation was treated as a rigid molecule and then the energy was calculated—that is, by rigid scanning. The PES scan was executed by the stepwise of the rotation of torsion angle for the initial molecule of GSK744 at the ωB97XD/6-31G* level in the Gaussian 09 package [50]. In detail, it was based on the dihedral angle (F(1)-F(2)-(O1)-(O2)) from −50° to 310° with an interval step of 5°. This approach can be applicable in the molecule with less flexible internal degrees of freedom. 

### 3.2. MOLPAK Global Search of Structures

In this work, MOLPAK software was used for the global search of crystal structures with the lowest lattice energies from 27 common space groups, including *P*1, *P*-1, *P*2, *Pm*, *Pc*, *P*21, *P*2/*c*, *P*21/*m*, *P*2/*m*, *P*21/*c*, *Cc*, *C*2, *C*2/*c*, *Pnn*2, *Pba*2, *Pnc*2, *P*221, *Pmn*21, *Pma*2, *P*21212, *P*212121, *Pca*21, *Pna*21, *Pnma*, *Fdd*2, *Pbcn*, and *Pbca*. The MOLPAK software [26] is based on the unique orientation of central molecules and constructs an approximate coordination pattern of the related molecules. A global search algorithm was designed to search for the filling mode having the smallest minimum bulk density of molecules with a fixed conformation. By rotating the central molecule with each step of 5° ranging from –90° to 90° in the three Cartesian planes, all unique stacking motifs of the target molecule were obtained. More than 3000 hypothetical dense candidates were identified with the space groups of *P*2_1_/*c*, *P*-1, *C*2/*c*, *Pbca*, *P*2_1_2_1_2_1_, etc. The predicted structures were subjected to preliminary calculations of lattice energy and density based on the built-in PMIN program with the repulsion-only UMD potential in the MOLPAK package [26]. The refined unique hypothetical structures by the PMIN program were ranked based on the minimum cell volume of each molecule and further carried out the minimization of their lattice energies with the repulsion–dispersion potential field. The 24 candidates, coming from five common space groups (*P*2_1_/*c*, *P*-1, *C*2/*c*, *Pbca*, and *P*2_1_2_1_2_1_), proceeded to the next round of Gibbs free energy calculations. The structure optimizations and free energy calculations were performed by our in-house parallel execution code.

### 3.3. Calculation of Gibbs Free Energy Parallel Execution Program

The proposed Gibbs free energy calculations were performed by the embedded fragment QM method, along with the DFT or the high-level second-order Møller–Plesset perturbation (MP2) theory, depending on the sizes of the target molecules. Considering the computational accuracy and time, we used the embedded fragment quantum mechanics method [38,39], which was performed by our in-house parallel execution code, to calculate the total energy of a unit cell and optimize the crystal structure at the ωB97XD/6-31G* level. The unit cell expanded into a supercell (4 × 4 × 4) with the periodic boundary condition and then we considered the two-body QM interaction if the distance of any two fragments in the supercell was less than or equal to a predefined cutoff distance (λ). The internal energy (*E_e_*) of a unit cell can be calculated by Equation (1):

(1) $$E_{e}=\underset{i}{\sum }E_{i\left(0\right)}+\underset{\begin{array}{c} i,j,i<j\\ R_{ij}\leq \lambda \end{array}}{\sum }\left(E_{i\left(0\right)j\left(0\right)}-E_{i\left(0\right)}-E_{j\left(0\right)}\right)\;+\frac{1}{2}\sum_{N=-S}^{S}\left(1-\delta_{n0}\right)\sum_{\begin{array}{c} i,j\\ R_{ij}\leq \lambda \end{array}}\left(E_{i\left(0\right)j\left(n\right)}-E_{i\left(0\right)}-E_{j\left(n\right)}\right)+E_{LR}$$

where λ is the distance threshold (λ is set to 4.0 Å), *n* is the three-integer index of a unit cell, and *R_ij_* is the distance between molecules *i* and *j*. *E_i(0)j(n)_* is the energy of the dimer for the *i*th molecule in the central unit cell and the *j*th molecule in the *n*th unit cell. The enthalpy (*H_e_*) per unit cell can be calculated using Equation (2):

(2) $$H_{e}=E_{e}+PV$$

where *V* is the unit cell volume, and *P* is the external pressure. The convergence criterion of geometry optimization is set to 0.001 Hartree/Bohr for the maximum gradient.

The Gibbs free energy (*G_e_*) of a unit cell can be calculated using Equation (3):

(3) $$G_{e}=H_{e}+U_{v}-TS_{v}$$

where *T* is the temperature, *Uv* the zero-point vibrational energy, and *Sv* the entropy per unit cell. For molecular crystals, the zero-point energy *Uv* and entropy *Sv* can be obtained by Equations (4) and (5) with the harmonic approximation:

(4) $$U_{v}=\frac{1}{K}\sum_{n}\sum_{k}\omega_{nk}\left(\frac{1}{2}+\frac{1}{e^{\beta \omega_{nk}}-1}\right)$$

(5) $$S_{v}=\frac{1}{\beta TK}\sum_{n}\sum_{k}\left\{\frac{\beta \omega_{nk}}{e^{\beta \omega_{nk}}-1}-\ln\left(1-e^{-\beta \omega_{nk}}\right)\right\}$$

where *ω*_*n***k**_ is the frequency of the phonon in the *n*th phonon branch with the wave vector **k**, and *β = 1/k_B_T*; *k_B_* is the Boltzmann constant. The product over **k** must be taken over all *K* evenly spaced grid points of **k** in the reciprocal unit cell. In this study, a **k**-grid of 21 × 21 × 21 was used (*K* = 9261).

## 4. Conclusions

In summary, this work integrated high-precision methods for the prediction of crystal structure and morphology of molecules, as a case study of the HIV integrase inhibitor cabotegravir (GSK744). Combining MOLPAK prediction and Gibbs free energy calculation, we presented the crystal prediction of cabotegravir and demonstrated that Str. 14 is the most likely stable structure. Even in a pool of predicted candidate structures lacking the ones closely presenting the real structure, Gibbs free energy still outperformed the conventional lattice energy by targeting the most promising candidate for the further refinement. This work reveals the success and remaining challenge of conventional crystal structure prediction and underscores the unique value of Gibbs free energy in guiding further structural refinement. The integration of Gibbs free energy could inspire novel structural refinement approaches in the future. The crystal stabilities of GSK744 structures were evaluated based on Gibbs free energy calculations. We also expect that the crystal structure of GSK744 determined in this work will become a valuable resource for the further engineering of nanomedicines of this important HIV therapeutic and PrEP drug.

## Data Availability

The data presented in this study are available from the corresponding author upon reasonable request.

## Supplementary Materials

The following are available online, Table S1: The lattice parameters of 24 selected GSK744 candidates from 3000 predicted structures by MOLPAK software, Figure S2: Crystal structure of Str. 14.
