# Supplementary material for: Quantum Mechanical-Based Stability Evaluation of Crystal Structures for HIV-Targeted Drug Cabotegravir
Source: Molecules. 2021 Nov 26;26(23):7178. doi: 10.3390/molecules26237178 (PMC8659202; doi:10.3390/molecules26237178)
Supplement: Supplementary file 1 [file molecules-26-07178-s001.zip › molecules-1478496-supplementary.pdf]

molecules

Supplementary Materials

Quantum Mechanical-Based Stability Evaluation of Crystal Structures for HIV-Targeted Drug Cabotegravir

Yanqiang Han[1,2†], Hongyuan Luo[2†], Qianqian Lu[2†], Zeying Liu[1], Jinyun Liu[3*], Jiarui Zhang[4], Zhiyun Wei[1*] and Jinjin Li[1,2*]

[1]Shanghai Key Laboratory of Maternal Fetal Medicine, Shanghai First Maternity and Infant Hospital, School of Medicine, Tongji University, Shanghai 200092, China; hanyanqiang@sjtu.edu.cn (Y.H.); 2031246@tongji.edu.cn (Z.L.); zhiyun_wei@163.com (Z.W.); lijinjin@sjtu.edu.cn (J.L.)
[2]Key Laboratory for Thin Film and Microfabrication of Ministry of Education, Department of Micro/Nano-electronics, Shanghai Jiao Tong University, Shanghai 200240, China; hanyanqiang@sjtu.edu.cn (Y.H.); joker_luo@sjtu.edu.cn (H.L.); luqianqian_studying@sjtu.edu.cn (Q.L.); lijinjin@sjtu.edu.cn (J.L.)
[3]Key Laboratory of Functional Molecular Solids of the Ministry of Education, Anhui Provincial Engineering Laboratory for New-Energy Vehicle Battery Energy-Storage Materials, School of Chemistry and Materials Science, Anhui Normal University, Wuhu, Anhui 241002, China; jyliu@ahnu.edu.cn
[4]Division of Computational Biomedicine, Boston University School of Medicine, Boston, MA 02118, USA; zjr@bu.edu
* Correspondence: jyliu@ahnu.edu.cn (J.L.); zhiyun_wei@163.com (Z.W.); lijinjin@sjtu.edu.cn (J.L.)
†These authors contributed equally to this work.

1. The Predicted Candidates for GSK 744

The crystal structure prediction (CSP) is performed by MOLPAK software on GSK744 molecule and generates more than 3,000 predicted structures. We select 24 candidates with the lowest lattice energies and list their lattice parameters in Table S1. From Table S1, the lattice energies for the 24 candidates range from -175.662 to -184.098 kJ/mol, corresponding to the structures of Str. 24 and Str.1, respectively. The selected 24 candidates have the similar density and lattice constants, but with different space groups. From Table S1, Str.1 has the lowest lattice energy (-184.098 kJ/mol), but we cannot speculate that Str.1 is the most thermodynamically stable structure for GSK744; further calculation and comparison of Gibbs free energy are needed.

Table S1. The lattice parameters of 24 selected GSK744 candidates from 3,000 predicted structures by MOLPAK software.

Nos.	$E_{lattice}$ (kJ/mol)	Density (g/cm)	Space Group	a(Å)	b(Å)	c(Å)	α(°)	β(°)	γ(°)
**Str. 1**	-184.098	1.325	$P2_1/c$	10.1445	13.6997	14.6216	90	90.981	90
**Str. 2**	-183.470	1.367	P-1	8.8954	9.449	13.4557	74.155	76.525	66.068
**Str. 3**	-183.424	1.372	C2/c	5.0472	18.7029	22.6978	90	112.46	90
**Str. 4**	-183.331	1.382	Pbca	9.5552	13.1076	31.1101	90	90	90
**Str .5**	-182.782	1.318	$P2_1/c$	8.3406	14.3224	17.4757	90	101.998	90
**Str. 6**	-182.489	1.388	P-1	7.5297	7.6706	16.947	88.457	84.104	85.048
**Str .7**	-182.345	1.338	Pbca	8.3688	18.1171	26.5489	90	90	90
**Str. 8**	-182.298	1.385	C2/c	7.5766	14.8226	17.7152	90	102.348	90
**Str. 9**	-181.838	1.398	$P2_12_12_1$	5.1764	18.7547	19.8361	90	90	90
**Str. 10**	-181.796	1.37	Pbca	10.0016	12.3242	31.8979	90	90	90
**Str. 11**	-181.708	1.384	P-1	7.4042	8.4175	15.7947	90.884	95.275	96.871
**Str. 12**	-180.954	1.329	$P2_1/c$	7.2553	13.3904	20.8459	90	90.051	90
**Str. 13**	-180.871	1.402	Pbca	11.796	15.2713	21.3278	90	90	90
**Str. 14**	-180.494	1.372	$P2_12_12_1$	7.1675	8.2109	33.3487	90	90	90
**Str. 15**	-179.824	1.347	$P2_12_12_1$	7.3687	12.2934	22.0606	90	90	90

**Str. 16**	-178.401	1.384	P-1	7.3922	7.8241	17.3922	93.728	90.068	104.267
**Str. 17**	-177.773	1.387	$P2_12_12_1$	6.1521	10.3168	30.5822	90	90	90
**Str. 18**	-176.973	1.296	Pbca	7.9533	13.4868	38.7274	90	90	90
**Str. 19**	-176.801	1.347	C2/c	7.6484	7.6632	39.4002	90	89.64	90
**Str. 20**	-176.424	1.358	$P2_12_12_1$	5.0989	19.1673	20.2791	90	90	90
**Str. 21**	-176.211	1.328	$P2_12_12_1$	8.9686	9.9299	22.7605	90	90	90
**Str. 22**	-176.127	1.344	C2/c	7.6146	7.6523	39.818	90	106.28	90
**Str. 23**	-175.708	1.417	Pbca	9.3305	12.4028	32.8458	90	90	90
**Str. 24**	-175.662	1.324	$P2_1/c$	8.8354	13.2895	17.731	90	102.436	90

2. The Crystal Structure of Str. 14 in PDB Format

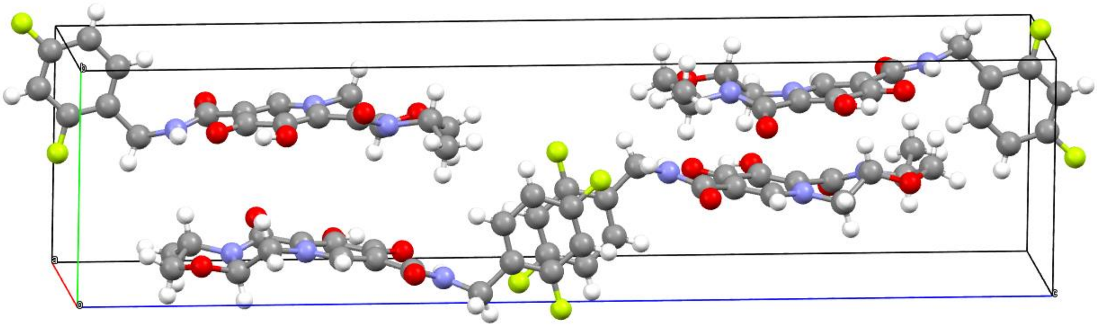

Figure S2. crystal structure of Str. 14.

The crystal structure of Str. 14 in PDB format:

CRYST1 7.168 8.211 33.349 90.00 90.00 90.00 P212121

ORIGX1 1.000000 0.000000 0.000000 0.00000

ORIGX2 0.000000 1.000000 0.000000 0.00000

ORIGX3 0.000000 0.000000 1.000000 0.00000

SCALE1 0.139519 0.000000 0.000000 0.00000

SCALE2 0.000000 0.121789 0.000000 0.00000

SCALE3 0.000000 0.000000 0.029986 0.00000

ATOM 1 C1 UNK C 1 4.671 0.753 3.832 1.00 0.00 C

ATOM 2 C2 UNK C 1 3.360 1.410 4.227 1.00 0.00 C

ATOM 3 H3 UNK C 1 3.421 2.396 4.071 1.00 0.00 H

ATOM 4 C4 UNK C 1 2.146 0.852 3.493 1.00 0.00 C

ATOM 5 O5 UNK C 1 1.030 1.138 4.358 1.00 0.00 O

ATOM 6 C6 UNK C 1 1.489 0.840 5.656 1.00 0.00 C

ATOM 7 H7 UNK C 1 1.340 -0.125 5.873 1.00 0.00 H

ATOM 8 N8 UNK C 1 2.930 1.154 5.621 1.00 0.00 N

ATOM 9 C9 UNK C 1 3.614 1.578 6.705 1.00 0.00 C

ATOM 10 O10 UNK C 1 4.772 1.989 6.668 1.00 0.00 O1-

ATOM 11 C11 UNK C 1 2.853 1.489 8.003 1.00 0.00 C

ATOM 12 C12 UNK C 1 3.472 1.343 9.210 1.00 0.00 C

ATOM	13	C13	UNK	C	1	2.724	1.221	10.452	1.00	0.00	C
ATOM	14	O14	UNK	C	1	3.387	1.070	11.513	1.00	0.00	O1-
ATOM	15	C15	UNK	C	1	1.296	1.272	10.332	1.00	0.00	C
ATOM	16	C16	UNK	C	1	0.731	1.430	9.089	1.00	0.00	C
ATOM	17	N17	UNK	C	1	1.456	1.526	7.964	1.00	0.00	N
ATOM	18	C18	UNK	C	1	0.795	1.728	6.660	1.00	0.00	C
ATOM	19	C19	UNK	C	1	0.382	1.052	11.494	1.00	0.00	C
ATOM	20	O20	UNK	C	1	-0.848	1.191	11.407	1.00	0.00	O1-
ATOM	21	N21	UNK	C	1	0.981	0.658	12.626	1.00	0.00	N
ATOM	22	C22	UNK	C	1	0.223	0.267	13.805	1.00	0.00	C
ATOM	23	C23	UNK	C	1	0.176	1.346	14.864	1.00	0.00	C
ATOM	24	C24	UNK	C	1	0.174	2.695	14.551	1.00	0.00	C
ATOM	25	C25	UNK	C	1	0.110	3.666	15.530	1.00	0.00	C
ATOM	26	C26	UNK	C	1	0.052	3.263	16.845	1.00	0.00	C
ATOM	27	C27	UNK	C	1	0.064	1.951	17.225	1.00	0.00	C
ATOM	28	C28	UNK	C	1	0.120	1.023	16.210	1.00	0.00	C
ATOM	29	F29	UNK	C	1	0.120	-0.286	16.556	1.00	0.00	F
ATOM	30	F30	UNK	C	1	-0.031	4.201	17.821	1.00	0.00	F
ATOM	31	O31	UNK	C	1	4.810	1.234	9.317	1.00	0.00	O
ATOM	32	H32	UNK	C	1	4.591	-0.219	3.929	1.00	0.00	H
ATOM	33	H33	UNK	C	1	4.879	0.973	2.900	1.00	0.00	H
ATOM	34	H34	UNK	C	1	5.389	1.081	4.413	1.00	0.00	H
ATOM	35	H35	UNK	C	1	2.032	1.294	2.615	1.00	0.00	H
ATOM	36	H36	UNK	C	1	2.239	-0.123	3.351	1.00	0.00	H
ATOM	37	H37	UNK	C	1	-0.216	1.474	9.024	1.00	0.00	H
ATOM	38	H38	UNK	C	1	0.865	2.676	6.385	1.00	0.00	H
ATOM	39	H39	UNK	C	1	-0.164	1.490	6.723	1.00	0.00	H
ATOM	40	H40	UNK	C	1	1.861	0.634	12.657	1.00	0.00	H
ATOM	41	H41	UNK	C	1	-0.702	0.042	13.533	1.00	0.00	H
ATOM	42	H42	UNK	C	1	0.630	-0.548	14.194	1.00	0.00	H
ATOM	43	H43	UNK	C	1	0.217	2.958	13.640	1.00	0.00	H
ATOM	44	H44	UNK	C	1	0.108	4.589	15.303	1.00	0.00	H
ATOM	45	H45	UNK	C	1	0.036	1.692	18.138	1.00	0.00	H
ATOM	46	H46	UNK	C	1	5.028	1.253	10.128	1.00	0.00	H
TER	47										
CONECT	1	2	34	32	33						
CONECT	2	1	3	4	8						
CONECT	3	2									
CONECT	4	2	35	36	5						
CONECT	5	4	6								
CONECT	6	5	18	8	7						
CONECT	7	6									

```
CONECT    8    2    6    9
CONECT    9    8   10   11
CONECT   10    9
CONECT   11    9   17   12
CONECT   12   11   13   31
CONECT   13   12   15   14
CONECT   14   13
CONECT   15   13   16   19
CONECT   16   15   37   17
CONECT   17   11   16   18
CONECT   18    6   17   39   38
CONECT   19   15   20   21
CONECT   20   19
CONECT   21   19   22   40
CONECT   22   21   41   23   42
CONECT   23   22   24   28
CONECT   24   23   25   43
CONECT   25   24   44   26
CONECT   26   25   27   30
CONECT   27   26   28   45
CONECT   28   23   27   29
CONECT   29   28
CONECT   30   26
CONECT   31   12   46
CONECT   32    1
CONECT   33    1
CONECT   34    1
CONECT   35    4
CONECT   36    4
CONECT   37   16
CONECT   38   18
CONECT   39   18
CONECT   40   21
CONECT   41   22
CONECT   42   22
CONECT   43   24
CONECT   44   25
CONECT   45   27
CONECT   46   31
END
```
